# The largest subunit of RNA polymerase II from the Glaucocystophyta: functional constraint and short-branch exclusion in deep eukaryotic phylogeny

**DOI:** 10.1186/1471-2148-5-71

**Published:** 2005-12-09

**Authors:** John W Stiller, Leslie Harrell

**Affiliations:** 1 Department of Biology, Howell Science Complex, N108, East Carolina University, Greenville, NC USA

## Abstract

**Background:**

Evolutionary analyses of the largest subunit of RNA polymerase II (RPB1) have yielded important and at times provocative results. One particularly troublesome outcome is the consistent inference of independent origins of red algae and green plants, at odds with the more widely accepted view of a monophyletic Plantae comprising all eukaryotes with primary plastids. If the hypothesis of a broader kingdom Plantae is correct, then RPB1 trees likely reflect a persistent phylogenetic artifact. To gain a better understanding of RNAP II evolution, and the presumed artifact relating to green plants and red algae, we isolated and analyzed *RPB*1 from representatives of Glaucocystophyta, the third eukaryotic group with primary plastids.

**Results:**

Phylogenetic analyses incorporating glaucocystophytes do not recover a monophyletic Plantae; rather they result in additional conflicts with the most widely held views on eukaryotic relationships. In particular, glaucocystophytes are recovered as sister to several amoebozoans with strong support. A detailed investigation shows that this clade can be explained by what we call "short-branch exclusion," a phylogenetic artifact integrally associated with "long-branch attraction." Other systematic discrepancies observed in *RPB*1 trees can be explained as phylogenetic artifacts; however, these apparent artifacts also appear in regions of the tree that support widely held views of eukaryotic evolution. In fact, most of the RPB1 tree is consistent with artifacts of rate variation among sequences and co-variation due to functional constraints related to C-terminal domain based RNAP II transcription.

**Conclusion:**

Our results reveal how subtle and easily overlooked biases can dominate the overall results of molecular phylogenetic analyses of ancient eukaryotic relationships. Sources of potential phylogenetic artifact should be investigated routinely, not just when obvious "long-branch attraction" is encountered.

## Background

Evolutionary analyses of RNA polymerases, and RNA polymerase II (RNAP II) in particular, have provided important phylogenetic inferences about ancient evolution. The RNAP largest subunit has played a key role in resolving such widely accepted hypotheses as the three domains of life [1,2] and putative affiliation of the "long-branch" Microsporidia with fungi [3]; however, one particular inference of eukaryotic relationships based upon the RNAP II largest subunit (RPB1) has proven controversial. *RPB*1 sequences consistently recover a polyphyletic kingdom Plantae, with independent origins of red algae and green plants [4-9]. This result is in conflict with a growing consensus on eukaryotic relationships from other molecular phylogenetic analyses (see [10] for review).

The hypothesis that red algae are related closely to green algae and plants grew out of sequence-based phylogenetic analyses of plastid-based characters (see [11] and [12] for seminal early reviews). A monophyletic association of most plastid-based molecular characters lent support to the hypothesis of a single plastid origin [13]. Because both red algae and green plants have "primary" plastids (thought to be descended directly from a cyanobacterial endosymbiont) it is reasonable to assume that plastids originated in the common host cell ancestor of the two groups [12]. Although these data also can be reconciled with polyphyletic plastid origins [14,15], analyses of a number of nuclear genes likewise recover a monophyletic association of the red and green host cell lines [7,16] (but see [17] for alternative result). Congruence among a number of molecular phylogenies, from both host cell and plastid-based characters, has led to general acceptance of the hypothesis that all photosynthetic eukaryotes with primary plastids share a common ancestor [18-21]. This consensus view of plant evolution even has been incorporated into the phylogenetic treatment of eukaryotes in major biology textbooks [22-24]. Consequently, a polyphyletic Plantae recovered in *RPB*1 analyses typically is interpreted as a phylogenetic artifact [13,16,19,20].

As part of a general investigation of RNAP II evolution and function, we have examined this persistent phylogenetic conflict between *RPB*1 and other molecular analyses. A key taxon missing from previous *RPB*1 surveys was the Glaucocystophyta, a small, enigmatic group of photosynthetic protists also believed to harbor primary plastids [25-27]. Although relatively uncommon in nature [28,29], glaucocystophytes have intrigued phycologists and evolutionary biologists for over a century because of their cyanelles, photosynthetic organelles with characteristics intermediate between those of derived plastids and cyanobacteria. Historically, the pigments and vestigial peptidoglycan cell wall of cyanelles were taken as evidence of an intermediate relationship between the glaucocystophyte host cell and more recently acquired endosymbiont [29]. Current views hold that cyanelles and plastids have descended from the same endosymbiotic cyanobacterial ancestor [18-20,27], and phylogenetic analyses of large, multi-gene plastid and nuclear data sets both provide strong support for a monophyletic association of glaucocystophytes with red algae and green plants [21]. As the potential "missing link" in the evolution of primary eukaryotic photosynthesis, glaucocystophytes could provide ancestral data for clarifying the origins of red and green plants and overcoming phylogenetic artifacts that produce conflicts among molecular data.

We sequenced the complete RPB1 gene from *Glaucocystis nostochinearum *Itz., including the region encoding the C-terminal domain (CTD), as well as a partial sequence from *Cyanophora paradoxa *Korsh. Here we report comparative analyses of inferred protein sequences from these two species and a broad sample of other eukaryotes in an effort to understand the overall topology of the RPB1 tree, and the specific branching positions of green plants, red algae and glaucocystophytes.

## Results and Discussion

### Characterization of *RPB*1 from Glaucocystophytes

Most molecular analyses of the Glaucocystophyta have focused on *Cyanophora*; therefore, we made an effort to recover *RPB*1 from it and *Glaucocystis*. We encountered several technical problems, however, in our attempts to sequence the complete gene from *Cyanophora*. First, a persistent PCR artifact occurred with 3' RACE (Rapid Amplification of cDNA Ends), preventing direct recovery of sequence distal to conserved region G [30]. In addition, we identified two distinct *RPB*1 sequences from *Cyanophora*. Although they differ only at synonymous positions, the presence of two sequences complicated efforts to isolate a single contiguous gene product through standard RT (reverse transcription) and PCR methods. Therefore, we concentrated on recovering the complete RPB1 gene from *Glaucocystis*.

The most interesting overall feature of *Glaucocystis RPB*1 is that it encodes a typical RNAP II C-terminal domain (CTD). In its canonical form, the CTD comprises tandemly repeated heptapeptides with the consensus sequence Y_1_-S_2_-P_3_-T_4_-S_5_-P_6_-S_7 _[31]. These heptapeptides act as a platform for various proteins functionally associated with RNAP II transcription. CTD-protein interactions help regulate gene expression, couple transcription to pre-mRNA processing and post-transcriptional silencing, and generally coordinate nuclear function [32-35]. The CTD is missing or degenerate in many eukaryotic groups, but is conserved across the broad diversity of animals and fungi, as well as their putative protistan ancestors [4]. This strong conservation is not surprising, given numerous and essential CTD functions in mRNA synthesis.

Although its biochemical interactions are not as well-characterized as in animals and yeast, the CTD also is present in all green algae and plants examined to date [36]; based on comparative genomic analyses, core CTD-protein interactions also appear to be conserved across all of these groups [37]. Given this strong conservation of CTD form and function, it is reasonable to conclude that the protistan ancestor of green plants and algae also used CTD-based RNAP II transcription. In this light, the presence of a CTD in glaucocystophytes is consistent with the hypothesis that they share a common ancestor with green plants, and lends support to a broader kingdom Plantae including other eukaryotes with primary plastids. By the same token, the most straightforward explanation for the absence of a conserved CTD in most red algae [38] is that rhodophytes do not share a common ancestor with green plants and glaucocystophtyes.

As discussed previously, phylogenetic analyses of *RPB*1 sequences likewise have indicated that red algae originated independently of a common ancestor of green plants, fungi, animals and related protists. It is precisely in these latter eukaryotic groups that the CTD is invariably conserved, suggesting that CTD-based RNAP II transcription was canalized in their common ancestor [4]. If the now widely accepted hypothesis of a monophyletic Plantae is accurate, then both a "CTD-clade" and the independent origin of red algae inferred from RPB1 sequences must result from a tree-building artifact. A recent genome-level investigation of the CTD and its attendant proteins provides an explanation for just such an artifact: the CTD-clade recovered in RPB1 phylogenies reflects parallel functional constraints on RNAP II and related proteins, rather than historical signal retained in their sequences [37]. If true, then the polyphyly of green plants and red algae represents a phylogenetic artifact of sequence covariation [39] resulting from selection for differing mechanics of RNAP II transcription among eukaryotic lineages. The inclusion of glaucocystophyte sequences in RPB1 analyses might provide ancestral information that could help overcome such an artifact.

### Phylogenetic analyses of RPB1 sequences

**Figure 1:**
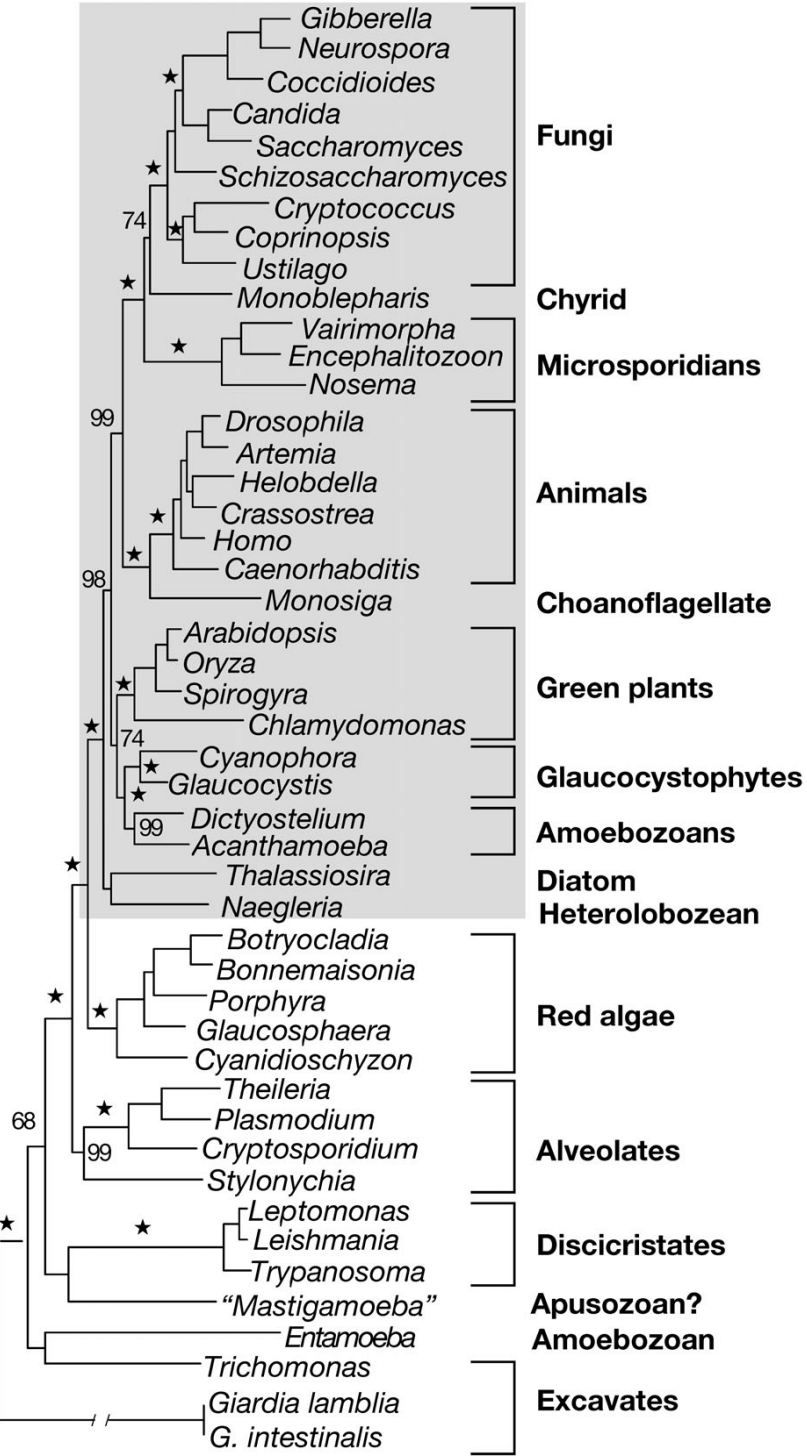
Tree recovered by ML (JTT + Γ + I) using alignment of 47 RPB1 sequences. Bayesian inference (also JTT + Γ + I) produced the same topology. Bayesian support values ( denotes 100%) are shown above or to the right of their respective nodes. CTD-clade is highlighted in gray. One half of the extremely long branch leading to *Giardia *has been removed for convenience. The taxonomic affinity of "*Mastigamoeba invertens*" has been unclear, but it now appears to be related to the proposed phylum Apusozoa [85] (Giselle Walker, personal communication). Branch lengths are from ML analysis.

The addition of glaucocystophyte RPB1 sequences does not yield a monophyletic Plantae. Both maximum-likelihood (ML) and Bayesian inference still recover a "CTD-clade" (Figure 1); it includes green plants and glaucocystophytes but not red algae. Even more problematic is an unexpected but strongly supported clade grouping glaucocystophytes with *Acanthamoeba *and *Dictyostelium*, members of the Amoebozoa [10,40,41]. To sample as broadly as possible, we included a number of partially sequenced genes (including *Cyanophora RPB*1) in our 47-taxon analysis; as a result, the alignment (available upon request) incorporates large blocks of missing data. In an effort to ameliorate potential sources of phylogenetic artifact, we aligned 30 of the most complete *RPB*1 sequences retaining multiple representatives of major lineages. We also excluded *Giardia *and the microsporidians. Although these sequences are complete, *Giardia *is the strongest source of "long-branch attraction" in *RPB*1 analyses [6,9]. Likewise, the microsporidians are a potentially significant source of phylogenetic artifact [3], particularly with respect to the *a priori *expectation that amoebozoans will associate with Opisthokonts (animals + fungi) [10].

**Figure 2:**
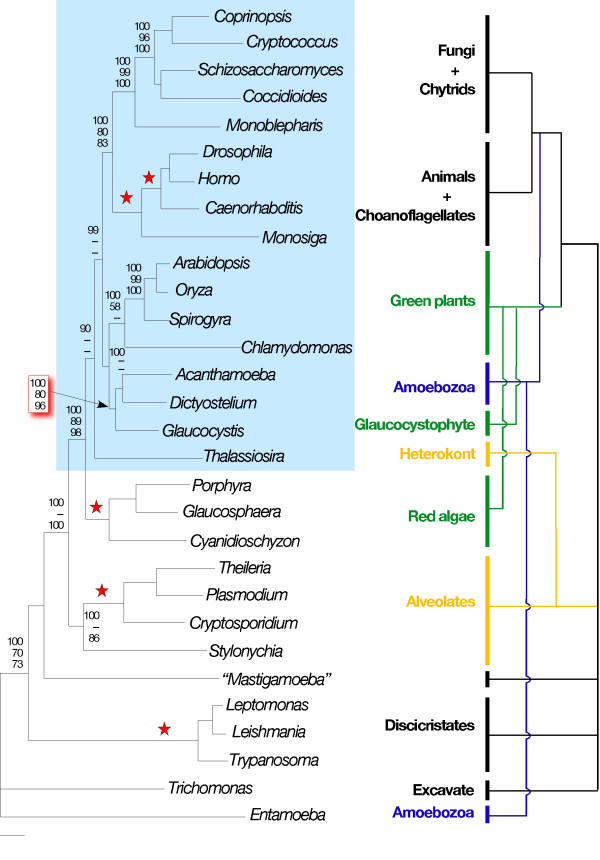
Tree recovered by both ML and Bayesian inference (both using JTT + Γ + I) from an alignment of 30 RPB1 sequences containing little or no missing data. Support values above or to the right of nodes indicate, from top to bottom, Bayesian inference, ML bootstrap, NJ bootstrap. Values supporting the *Glaucocystis*/*Acanthamoeba*/*Dictyostelium *clade are highlighted in red. Red stars indicate that all three values were 100% and dashes that the value was below 50%. The CTD-clade is highlighted in light blue. To the right is comparative phylogeny showing where the RPB1 tree disagrees with generally accepted views of eukaryotic phylogeny, following the review of Baldauf [10]. Those discrepancies are highlighted in color: green shows the hypothesis of a monophyletic kingdom Plantae, comprising all eukaryotes with primary plastids, yellow the "Chromalveolate hypothesis," and blue the hypothesis of a monophyletic Amoebozoa. Branch lengths are from ML analysis. The specific tree with branch lengths recovered by Bayesian inference is included as a supplement (see additional file 2).

Eliminating partial sequences and "long-branch" taxa has little effect on the tree topology. *Glaucocystis *still associates strongly with *Acanthamoeba *and *Dictyostelium *in Bayesian inference, ML and distance bootstrap analyses (Figure 2). This grouping also is recovered in parsimony analyses, but with low bootstrap support (see below). In addition, the "CTD-clade" is recovered using all four standard phylogenetic methods, although generally without strong support. This poses a number of problems with respect to leading hypotheses of eukaryotic relationships. *Entamoeba*, which has no CTD, is excluded from the CTD-clade containing other amoebozoans. The diatom *Thalassiosira *groups with CTD-containing taxa, not with ciliates and apicomplexans as predicted by the "Chromalveolate hypothesis" [42,43]. Finally, as noted above, red algae do not group with green plants and glaucocystophytes as predicted by the kingdom Plantae hypothesis. In fact, with this data set a monophyletic Plantae is rejected significantly in both KH and SH tests (*P *= 0.002 and 0.001 respectively); this appears to be due largely to the strong association of glaucocystophytes and amoebozoans, as support for a polyphyletic Plantae is reduced when *Acanthamoeba *and *Dictyostelium *are removed from the data set (KH, *P *= 0.054; SH, *P *= 0.007). We therefore undertook a detailed investigation to determine why *RPB*1 sequences generate such an unorthodox tree topology, beginning with the positions of *Glaucocystis*, *Dictyostelium *and *Acanthamoeba*.

### Why might some amoebozoans group with glaucocystophytes?

The strong association between the two amoebozoans and glaucocystophytes would appear to have one of three explanations: 1) they are, indeed, evolutionary sister groups; 2) their pairing reflects an ancient lateral gene transfer (LGT) of *RPB*1 from a glaucocystophyte to the common ancestor of *Acanthamoeba *and *Dictyostelium*; or 3) their association is a phylogenetic artifact. Although the first explanation cannot be rejected outright, molecular analyses usually group amoebozoans with animals and fungi [7,10,40], and we can find no consequential evidence (outside the *RPB*1 phylogeny presented here) to support a relationship between amoebae and glaucocystophytes. Thus, we presume that the *RPB*1 tree topology does not accurately reflect organismal relationships.

Likewise, given the number of co-adapted proteins interacting to form the RNAP II holoenzyme [44,45], not to mention associated general and specific transcription factors [46,47], LGT of the largest subunit seems exceedingly unlikely. These complications are only exacerbated if RPB1 anchors additional co-adapted CTD-protein interactions [32,34,35]. Moreover, a comparison of intron positions gives no indication of a glaucocystophyte ancestry for the *Acanthamoeba *RPB1 gene (*Dictyostelium RPB*1 contains no introns), nor are there any diagnostic indels to suggest such a relationship (alignment and intron data available upon request). Thus, with the exception of RPB1-based phylogenies, there is no evidence to suggest LGT between glaucocytophytes and amoebozoans. If conflicting gene phylogenies represent its only support, LGT is an unfalsifiable hypothesis. Any phylogenetic conflict can be resolved by invoking lateral transfer among the misbehaving taxa. Therefore, although neither of the first two hypotheses can be ruled out absolutely, we concentrated on the prospect that phylogenetic artifacts are responsible for the glaucocystophyte + amoebozoan grouping.

### Analyses of potential sources of phylogenetic artifacts

**Figure 3:**
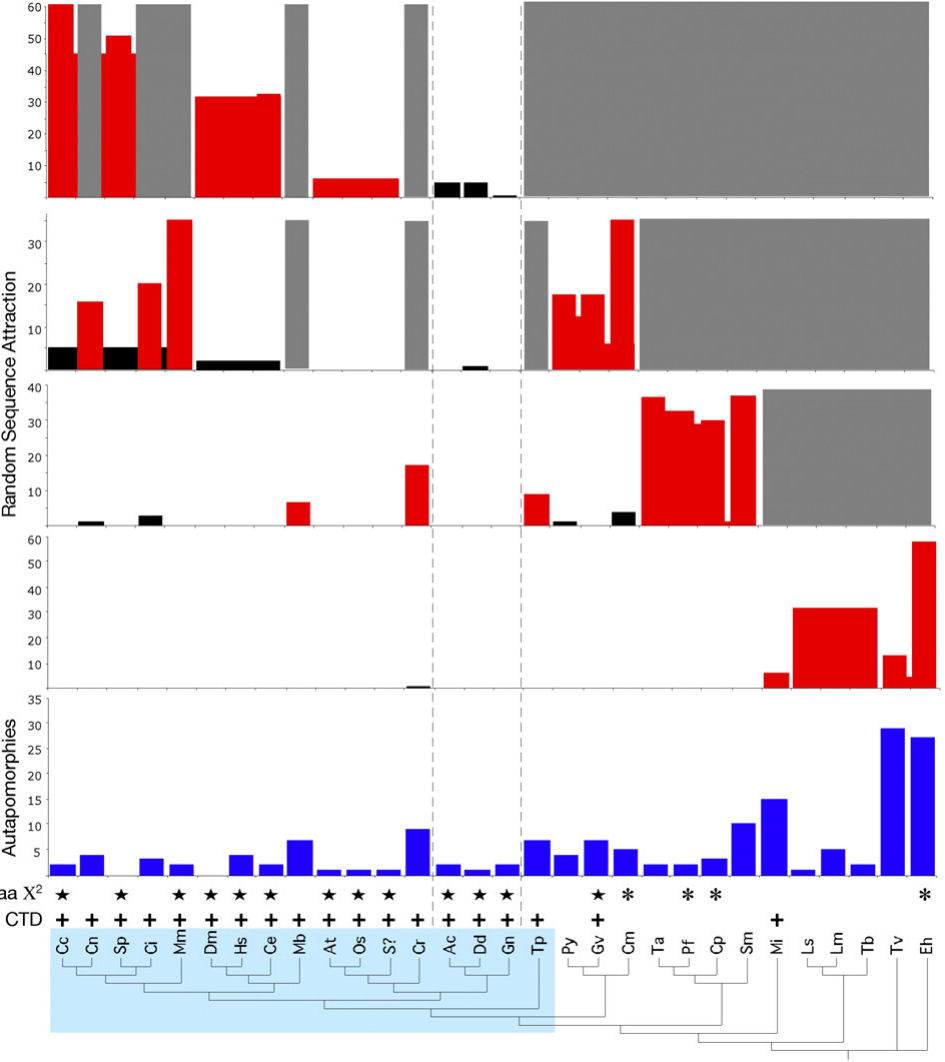
Analyses of indicators that could lead to phylogenetic artifacts in RPB1 sequences. The tree at the base is the same as shown in Figure 2, but without ML branch lengths. The CTD-clade is shaded in light blue. From bottom to top, the following are provided for each sequence. 1) **CTD: "+" **above a sequence indicates that it contains a C-terminal sequence that is consistent with known requirements for CTD function [36, 86, 87]. 2) **aa χ^2^: **results of ML analysis of deviation from mean amino acid composition for each sequence. A "*" indicates that the sequence deviates significantly (*P *< 0.05) from average composition. "" denotes a χ^2 ^*P *value > 0.9, indicating that the sequence deviates little from average composition. 3) **Autapomorphies: **Blue bars show the number of unique substitutions at sites under strong stabilizing selection across eukaryotic diversity. Numbers tend to be suppressed in taxa with multiple representatives, because synapomorphies uniting the group are not scored. For example, all kinetoplastids share a number of unique substitutions at otherwise invariable sites. If only one kinetoplastid were to be included, the number of autapomorphies would be comparable to Tv and Eh (data not shown, but see figure 5 for examples from animal, fungal and plant clades). 4) **Random Sequence Attraction: **Bars show the number of equally parsimonious trees on which each of 100 randomly generated sequences attached to specific RPB1 sequences in parsimony analyses. Red indicates a significant attraction to random sequences (in greater than 5% of parsimony replicates). Because many replicates produced more than one equally parsimonious tree, numbers do not add up to 100. A bar extending across multiple taxa indicates that the random sequence attached to the internode supporting that group (for example, the long branch leading to the three kinetoplastids). RPB1 sequences that were significant poles of attraction were removed from the alignment and the analysis repeated. Four separate analyses were performed, each with 100 randomly generated sequences. The lowest graph shows results using the entire data set, with decreasing numbers of sequences in the graphs above. Sequences shaded out in gray were removed based on significant "long-branch attraction" in the analysis shown immediately below.

Neither the *Glaucocystis *sequence, nor those of *Dictyostelium *and *Acanthamoeba*, deviate significantly from ML estimated mean amino acid frequencies (Figure 3). In fact, in χ^2 ^analysis for each of the three sequences, *P *was greater than 0.9, indicating that they deviate very little from overall mean frequencies. The majority of sequences in the alignment do not deviate significantly from the average, many also at *P *> 0.9 (designated by stars in Figure 3). Thus, biases in estimated amino acid composition are insufficient to account for the glaucocystophyte + amoebozoan clade.

A disproportionate number of unique substitutions (at sites under strong stabilizing selection throughout eukaryotic evolution) can provide *prima facie *evidence of an increased evolutionary rate independent of any presumed tree topology [48]. By this measure, *Glaucocystis *and the two amoebozoans are among the most slowly evolving sequences (Figure 3), although a number of others have accumulated comparably few substitutions at highly conserved sites. Nonetheless *Glaucocystis *displays the fewest unique substitutions of any monotypic representative of an ancient eukaryotic lineage (Figure 3). Thus, in terms of both amino acid composition and the accumulation of autapomorphies, RPB1 genes from *Glaucocystis *and the two amoebozoans have changed less from their ancestral sequences than have those of most other taxa.

To assess the empirical tendency of RPB1 sequences to attract "long branches," we examined the behavior of randomly generated sequences of average amino acid composition. With the alignment including all 30 taxa, none of 100 random sequences was attracted to *Glaucocystis*, *Acanthamoeba *or *Dictyostelium *in any most parsimonious tree recovered (Figure 3). When significant points of long-branch attraction (LBA) were removed from the alignment, these three sequences still did not attract randomly generated "long branches." In fact, even when only the 11 RPB1 genes least prone to attract "long branches" were retained in the analysis, *Glaucocystis*, *Acanthamoeba *and *Dictyostelium *still attracted the fewest randomly generated sequences. Remarkably, given that it is the sole representative of an ancient lineage, *Glaucocystis *attracted only one random sequence in all of the analyses performed, the fewest for any taxon in our investigation. Furthermore, *Glaucocystis* was the only monotypic representative to survive into the final round of random sequence addition (Figure 3).

The results of three separate analyses of "long-branch" indicators show that sequences of *Glaucocystis*, *Acanthamoeba *and *Dictyostelium *are highly unlikely to be drawn together by "long-branch attraction." Rather, they appear to be among the most slowly diverging RPB1 genes (Fig 3). What then accounts for their recovery as a strongly supported clade? The tendency to attract a randomly generated sequence correlates with how randomized a given sequence has become with respect to its phylogenetic relatives – in other words, how much it has diverged from its most recent shared ancestral sequence. In 100 tests using the complete *RPB*1 data set, as well as in previous investigations of other gene sequences [6,48], two random sequences included in an alignment always attract each other. Four-sequence simulated phylogenies yield comparable results for completely and partially randomized sequences [49], although sequences with an intermediate level of randomization can actually repel long branches under the conditions modeled.

In large trees with complex hierarchical structure, random sequences virtually never attach to individual members of a clade of closely related taxa, even when its members display accelerated substitution rates. For example, although randomly generated sequences attach to the long internode leading to kinetoplastids in 32% of parsimony replicates (Figure 3), none are attracted to any of three sequences individually. This tendency mirrors the accumulation of unique substitutions at otherwise strongly conserved sites (Figure 3) [6,48], further supporting random sequence attraction as a measure of relative sequence divergence. Therefore, if the *Glaucocystis *+ amoebozoans clade is indeed artifactual, it is probably because their genes are the ***least ***derived from their common ancestral sequence; that is, they cluster on the basis of shared, ancestral positions lost from other taxa. Their overall similarity excludes randomized sequences from attaching to an individual branch within the group; this apparently extends to other more divergent RPB1 sequences as well.

**Figure 4:**
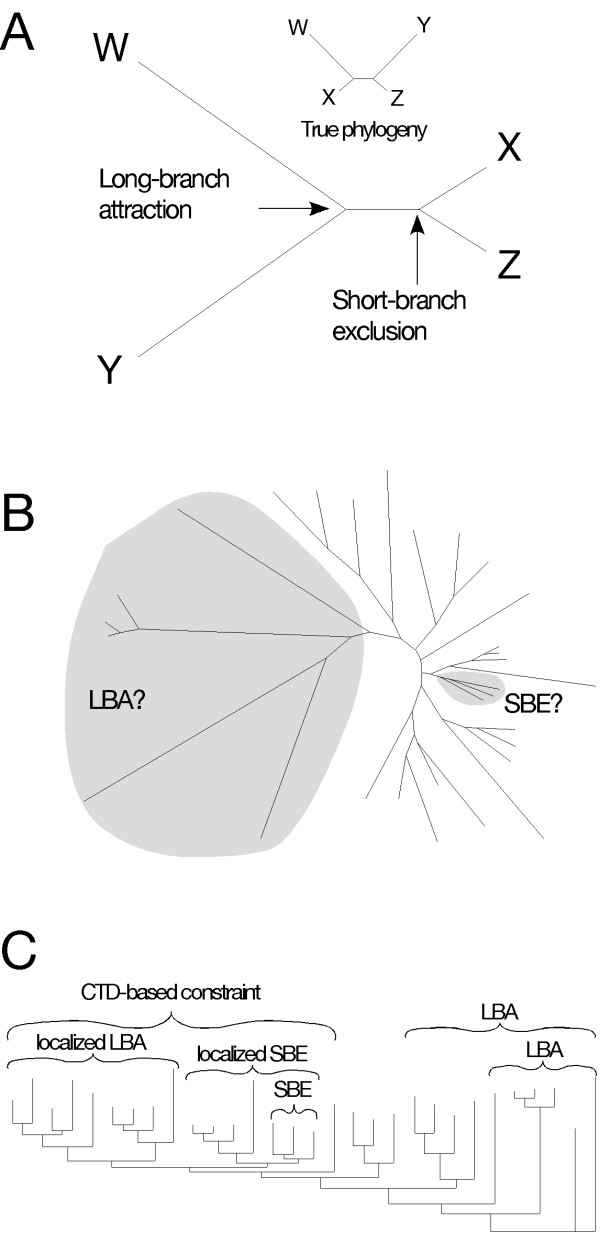
The appearance of phylogenetic artifacts in the RPB1 phylogeny due to "long-branch attraction" (LBA), "short-branch exclusion" (SBE), and CTD-based functional constraint on evolutionary change. **A. **Adapted from Felsenstein's original four taxon demonstration of "long-branch attraction" [66], this tree shows graphically the phenomena of LBA and SBE. A combination of large differences in substitution probabilities among branches, combined with short internodes, leads to artificial grouping of the more rapidly evolving sequences. This, by definition, results in an additional artificial grouping of shorter branches, at some level, which we call "short-branch exclusion." **B. **Unrooted phenogram of RPB1 tree from figure 2 more graphically demonstrating the large variation in inferred substitution probabilities in terminal branches, along with the generally short internodes throughout the tree. The four most basal lineages (as viewed in the rooted phenogram in figure 2) are consistent with a LBA artfifact, while the presumably artificial clustering of *Glaucocystis*, *Acanthamoeba *and *Dictyostelium *is most consistent with SBE. Both clades are highlighted in gray. **C. **Topological features of the global RPB1 tree that are consistent with the three kinds of artifacts discussed. With the complete data set, only the extreme long-branch features of the four most rapidly evolving basal sequences are obvious (Figure 3). With subsequent rounds of analysis, in each case removing the most long-branch sequences identified in the prior round, the branching pattern of subsections of the tree are shown to be consistent with one of the artifacts.

Although similar groupings have been uncovered with other molecular data sets [6,48,50], phylogenetic artifacts typically are viewed as "long-branch" effects resulting from the sequences that have experienced rapid or otherwise unusual modes of divergence [51]. As a result, these sequences are considered suspect, whereas those with lower than average rates typically are assumed to perform well in phylogenetic reconstruction. By definition, however, if a LBA artifact is present, then there also must be an artificial clustering of more slowly evolving taxa that should group with the respective long-branch sequences. We offer the phrase "short-branch exclusion" (SBE) to identify this associated artifact (Figure 4A). The SBE phenomenon uncovered in our analyses is consistent with demonstrated artifacts caused by differences in the proportion of variable sites (P_var_) across lineages [52]; this kind of complexity in rate variation can dominate tree-building signal in ancient phylogenetic reconstruction, including among sequences with low proportions of variable sites (that is, "slowly-evolving" taxa) [53]. The unexpected clustering of *Glaucocystis *and two amoebozoans, along with consistent evidence that the three are among the least diverged sequences in the analysis, give all the indications of such a "short-branch" artifact (Figure 4B).

### Phylogenetic artifacts and global tree topology

As noted above, the overall RPB1 tree topology and the specific positions of red algae, *Thalassiosira *and *Entamoeba *are consistent with recovery of a "CTD-clade," comprising all eukaryotic lineages in which the CTD has been strongly conserved while excluding those where it has been allowed to degenerate (Figures 2, 3). Originally this "CTD-clade" was hypothesized to be a natural group descended from a common ancestor in which CTD-based RNAP II transcription had coalesced [4,38]. More recent genome-level investigations of the CTD and its protein partners [37,54] indicate that the CTD-clade can be explained alternatively by parallel functional constraints in organisms that use CTD-based transcription, which lead to correlated patterns of *RPB*1 sequence evolution. Thus, the major discrepancies between the RPB1 tree and more widely accepted views of eukaryotic evolution (Figure 2) can be reconciled as artifacts of short-branch exclusion, and parallel or convergent evolution due to covariation in the mode of selection on the RPB1 molecule.

At first inspection it appears reassuring that analytical artifacts can explain apparent phylogenetic anomalies, specifically the recovery of a polyphyletic Plantae. Although red algal RPB1 genes are not particularly fast-evolving with respect to most eukaryotes, they exhibit greater "long-branch" tendencies than do sequences from other members of the hypothesized kingdom Plantae. Along with differing functional constraints on CTD-based RNAP II transcription, these subtle rate differences could explain the presumed artifact in RPB1 trees. Our investigation of "long-branch" indicators, however, raises a more general issue with respect to the global RPB1 tree; virtually the entire topology of the RPB1 tree is disturbingly consistent with those same sources of artifact. For example, if suspect and inconsistent tree-rootings are discounted, the branching position of alveolates is generally consistent with phylogenomic treatments [7,16]. In *RPB*1 analyses, this position is associated with a clade comprising the four most identifiable "long-branches," *Entamoeba*, *Trichomonas*, *Mastigamoeba*, and kinetoplastids. When the latter sequences are excluded, however, alveolates also display disproportionate long-branch tendencies (Figure 3). In effect, their branching position is consistent with a "long-branch attraction" artifact. Even within the CTD-clade – composed of sequences with the lowest rates and otherwise average patterns of divergence (Figure 3) – relationships among well-established groups are consistent with apparent rate variation among sequences.

**Figure 5:**
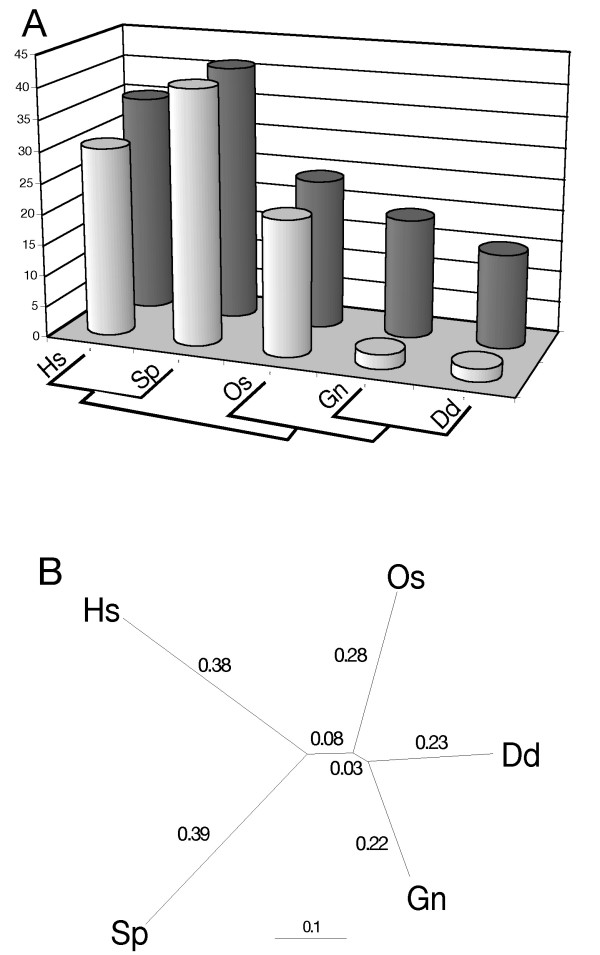
**A. **Analyses of long-branch indicators of *Glaucocystis*, and the most slowly evolving sequences of the animal, fungal, plant and amoebozoan clades based on analyses shown in figure 3. Lightly shaded bars show attachment of random sequences and darker bars unique substitutions at highly conserved sites. *Thalassiosira*, *Glaucosphaera *(the red alga with the least "long-branch" tendencies) and *Stylonychia *were included in the tabulation of unique substitutions (but not with random sequence analyses), to provide additional evidence that the sites in question were under strong stabilizing selection across eukaryotic diversity. With this sub-alignment, only single unique substitutions were scored. **B. **Five taxon ML tree (JTT + Γ + I) with branch lengths showing sequence change across branches and internodes. Unit is expected changes per amino acid position.

As a function of overall within-clade similarity, individual green plants and animals (with the exception of *Chlamydomonas*) do not attract random sequences, nor do they show an accumulation of unique substitutions (Figure 3). Behavior of the internodes leading to these clades, however, suggests that their individual sequences may represent somewhat "longer branches" than those of *Glaucocystis*, *Dictyostelium *or *Acanthamoeba *(Figure 3). Therefore, we analyzed unique substitutions and random sequence behavior using the representative sequence with the fewest "long-branch" tendencies from each group: human from animals, *Oryza *from plants, *Schizosaccarhomyces *from fungi, and *Dicytostelium *from amoebozoans. In this analysis, the relative short-branch tendencies of *Dictyostelium *and *Glaucocystis *become even more pronounced (Figure 5A), and their clustering is consistent with an SBE artifact (Figure 5B). Moreover, the green plant *Oryza*, recovered as sister to the *Glaucocystis*/*Dictyostelium *clade, has the next fewest "long-branch" indicators. The human + *Schizosaccharomyces *clade, which corresponds to the widely accepted systematic hypothesis of the Opisthokonta, then could be explained as a LBA artifact localized within a group of generally more slowly-evolving sequences. In model-based ML analyses, the branches leading to these two sequences have nearly twice the substitution-per-site probability of those for *Glaucocystis *and *Dictyostelium*, and five to ten times the probability of the two internodes that define overall branching order (Figure 5B).

**Figure 6:**
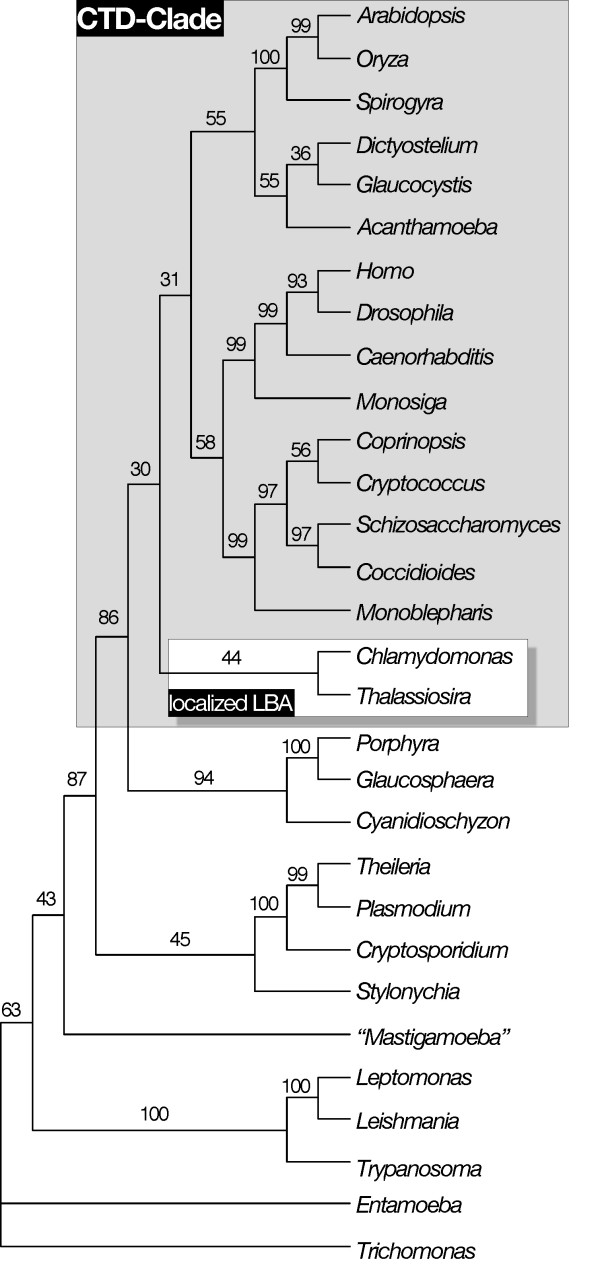
Parsimony tree based on RPB1 sequences showing a clear example of "localized LBA." The green alga *Chlamydomonas *groups with other green plants and algae in model-based approaches, but is attracted to the diatom *Thalassiosira *using parsimony. These sequences represent the two "longest branches" within the CTD clade, but do not share any other sub-clade affiliation in the RPB1 tree (Figure 2). This grouping demonstrates that LBA and SBE are not simply global effects, but can cause more localized artifacts as well. Note also that, in parsimony, *Glaucocystis *groups specifically with *Dictyostelium*, the shorter branched of the two amoebozoans (Figures 3, 5).

Direct evidence that such localized LBA can occur in phylogenetic reconstruction is immediately apparent in parsimony analyses of the RPB1 data set. Although it is a long-branch taxon compared to other green algae and plants, *Chlamydomonas *is placed correctly using likelihood and Bayesian algorithms (Figures 1, 2). In parsimony it falls victim to long-branch attraction. Rather than attaching to the strongest sources of LBA (*Entamoeba*, *Trichomonas*, kinetoplastids [see Figure 3]), however, *Chlamydomonas *is attracted to the diatom *Thalassiosira *(Figure 6) and the two emerge as the deepest branch of the CTD-clade. LBA pulls *Chlamydomonas *away from other green plant sequences, but unknown evolutionary constraints (apparently related to CTD-based transcription [37]) prevent it from being drawn completely out of the CTD-clade. Thus, the two longest branches that are constrained to fall within the CTD-clade attach to each other.

Generally it has been the case in sequence-based phylogenies that well-defined evolutionary lineages (green plants, animals, fungi, red algae, etc.) exclude other sequences and form strongly supported clades. This occurs even if a lineage has a generally high divergence rate (e.g. kinetoplastids in this study), so long as its members have not diverged too far from their common ancestral sequence. The challenge of deep molecular systematics has been to determine the relationships among these well-defined groups. When the potential for localized tree artifacts is considered, the overall relationships of these groups on the RPB1 tree are consistent with a combination of biases identified in the data. This is true even in those regions of the tree where sequences are undergoing relatively slow and comparable modes of evolution (Figure 5). In fact, the cumulative effects of artifacts can explain the entire backbone of relationships among major eukaryotic lineages (Figure 4C), and no signal from an historical pattern of relationships appears to be required. Given the number of putatively misplaced taxa (Figure 2), the implicit assumption that most regions of the tree reflect true evolutionary history is unwarranted.

### Broader implications for deep phylogenetics

The fact that a phylogeny is consistent with data biases does not exclude the possibility that the tree accurately reflects evolutionary history. It does say, however, that the null hypotheses cannot be rejected; that is, that random effects and/or data biases account for the pattern recovered (implicit in all phylogenetic analyses). Consequently, the alternative hypotheses that the tree is based on historical signal cannot be accepted.

It is possible that the RPB1 tree shown in figure 2 truly depicts the pattern of eukaryotic evolution. Given conflicts with other data sets, and the fact that much of its topology can be explained by rate variation and parallel constraint, it is more reasonable to conclude that the RPB1 tree is rife with phylogenetic artifacts. This assessment can be made because of accumulated data in three areas, which are unavailable for most sequences used in phylogenetic analyses of ancient evolution. First, RPB1 structure, function and biochemical interactions are well characterized, providing the framework for recognizing different functional constraints among taxa [37]. Second, extensive analyses of "long-branch" indicators have been performed, including for regions of the tree that do not appear to be subject to LBA by highly divergent sequences. Finally, topological incongruence exists between the RPB1 tree and more widely accepted hypotheses of eukaryotic relationships, providing an impetus to investigate specific discrepancies. Of course, in arguing that artifacts dominate RPB1 phylogenies we have assumed those broadly held hypothetical relationships to be true. Given the evidence of pervasive artifacts uncovered here, and in many other molecular phylogenetic studies of deep relationships as well [6,48,50,52,53,55-60], that assumption must be considered provisional.

Recent phylogenetic inferences of deep eukaryotic evolution have been made using large multi-gene data sets. The conclusions from these phylogenomic investigations have replaced an earlier model of global eukaryotic evolution based on small subunit ribosomal RNA sequences (SSU rDNA). At just about the time the SSU rDNA tree was adopted by major textbooks, it came under greater scrutiny largely due to developing conflicts with other molecular data sets [61-64]. Analyses of long-branch indicators demonstrated that the global topology of the rDNA tree was more consistent with variation in mode and tempo of evolution among sequences than with historical pattern [48]. The detailed analyses presented here suggest that the same is true of RPB1 sequences. Yet there is no reason to presume that these two genes are unusually prone to artifact.

As the gene encoding the largest subunit of RNAP II, *RPB*1 has the attributes of a reliable phylogenetic marker. It supplies a coding region of about 5 kb, over half of which consists of conserved domains that can be aligned reliably across most of eukaryotic diversity; this a relatively large data set for a single-gene phylogeny. It performs the same core function in all eukaryotes. There is no evidence that *RPB*1 has been carried as a multi-gene family over broad stretches of eukaryotic evolution, reducing the chance of paralogous sampling. Indeed, RPB1 phylogenetic analyses have been robust in the face of long-branch artifacts that plague microsporidian sequences in many other data sets [3], and parametric methods can overcome clearly identifiable phylogenetic artifacts that occur using parsimony (see discussion of *Chlamydomonas *above). Therefore, it is a reasonable to conclude that the biases found in *RPB*1 sequences are comparable to, if not less than, those present in most molecular markers. Indeed, Lockhart and colleagues [53] showed that changing distributions of sites that are variable and invariable can explain global tree topologies among major eubacterial lineages, suggesting that sequence-based phylogenies may provide little valid information about these ancient historical relationships.

## Conclusion

Although the subject has received increasing attention in recent years, phylogenetic investigations generally have operated under the assumption that tree-building artifacts are rare and restricted to odd and problematic taxa [51]. Implicit in phylogenomics is the assumption that the dominant overall tree-building signal from large, multi-gene alignments overcomes "noise" or biases that lead to conflicts between smaller data sets and, therefore, converges on true historical pattern. Indeed, this has been argued explicitly with respect to increasing support for a monophyletic Plantae as the number of genes included in the analysis grows [21]. Given both theoretical and empirical criteria, this assumption appears overly optimistic.

Biochemically-based models of sequence evolution predict that historical patterns should not be recoverable in phylogenetic analyses covering timescales on which the broad diversity of eukaryotes emerged [65]. Moreover, it has been demonstrated clearly that all phylogenetic algorithms can produce spurious outcomes when explicit or implicit model assumptions are violated (see [51] for thorough review); when violations result in statistical inconsistency, artifacts worsen as data sets increase in size [66,67]. Although parametric and probabilistic methods (such as ML and Bayesian inference) overcome parsimony artifacts under some conditions, they can actually under-perform parsimony when variation among rates at sites changes through time [68]. Presumably, complex patterns of sequence heterotachy and nonstationary covariation [39] have been the rule rather than exception over several billion years of eukaryotic evolution.

Covariation of parallel or convergent selection on functional constraints in sequence evolution has not been studied extensively, particularly with regard to its impact on phylogenetic analyses. This is for good reason; such covariation can be difficult to identify, even when the sequences in question (as in the case for RPB1) have relatively well-characterized functions and biochemical interactions [37]. Little to nothing is known about the functional interactions of most sequences used in phylogenomic investigations, nor can available phylogenetic methodologies yet compensate for such complex covariation, even when physical and biochemical constraints are known [39].

The indications of localized LBA and SBE uncovered in this investigation are subtle; they would be easy to miss, or to dismiss as too weak to affect tree topology. Nevertheless, they provide the most reasonable explanation for the aberrant grouping of glaucocystophyte and amoebozoan sequences. They must, therefore, be considered seriously with respect to other regions of the tree as well, including those that agree with expectations from prior molecular phylogenies. It is common in large phylogenomic treatments to remove overtly long-branch taxa to avoid tree-building artifacts, or to constrain "well-defined" groupings (such as the Opisthokonta or Plantae) to make computation more tractable [21,40]. These practices may well increase the impact of cryptic sources of covariation in the sequences retained.

There are serious conflicts among molecular data sets with respect to virtually all inferences about ancient eukaryotic relationships (e.g. [69,70]). This is true even for the most strongly supported and widely accepted hypotheses of relationships among eukaryotic lineages [15,71,72]. The overall lack of congruence of phylogenetic signal within genomes has prompted some researchers to question whether ancient relationships can be considered to be tree-like at all [73]. When two or more phylogenetic signals are present, there appears to be no basis for an *a priori *assumption that the dominant signal recovers historical relationships. Instead it may reflect parallel function or other constraints on sequence evolution that are difficult to detect. As molecular sequence data sets grow ever larger in size and complexity, it is critical that they be scrutinized thoroughly for potential biases that could affect phylogenetic inference; in particular, sequences with relatively slow apparent divergence rates should be examined carefully for evidence of short-branch exclusion. Finally, it is essential that alternative approaches to reconstructing evolutionary history continue to be explored.

## Methods

### Specimen preparation and nucleic acid extraction

An axenic culture of *C. paradoxa *(CCAC 0074) was obtained from the Culture Collection of Algae (CCAC) at the University of Cologne, Germany. Cells were grown in bubbling cultures of soil water medium with barley seeds (Carolina Biological, Burlington, NC) under constant fluorescent light at 25°C. *Glaucocystis nostochinearum *(UTEX-B 1929)was obtained from UTEX culture collection (Austin, TX) and grown under the same conditions, but in AlgaGro freshwater medium (Carolina Biological). Cells were pelleted in a table-top centrifuge and stored at -80°C for nucleic acid extraction.

*Glaucocysti*s samples were placed in a chilled mortar, flash frozen with liquid nitrogen, pulverized with a pestle to a fine powder and suspended in an equal volume of nucleic acid extraction buffer. Because *Cyanophora *lacks a cell wall, no grinding was required. DNA extractions were performed using a CTAB extraction method [74], with an additional purification using Qiagen mini-columns (Valencia CA). RNA was extracted with the Promega (Madison, WI) SV Total RNA Isolation System.

### Recovery of *RPB*1 sequences

GeneRacer RT-PCR (Invitrogen, Carlsbad, CA) was used to obtain the RPB1 coding regions from total RNA extractions, using universal degenerate primers [5,75]. Primers were used in nested pairs when necessary to amplify a recoverable DNA band. Since degenerate primers were involved, "touchdown" PCR was employed, with an annealing temperature ramped from 58 to 43°C over 15 cycles, followed by 25 cycles annealing at 55°C. The 5' end of the *RPB*1 transcript was obtained using RACE; mRNA was dephosphorylated, de-capped and ligated to a GeneRacer RNA oligo linker with nested priming sites, permitting selective recovery of messages complete on the 5' end. Linker primers were used in opposition to nested specific primers designed from sequences recovered previously using universal primers. To complete the 3' end of the gene, an oligo dT linker was used in RT-PCR in opposition to sequence specific primers from region G. To determine the number and position of introns, *RPB*1 was isolated from genomic DNA by PCR using overlapping sequence-specific primers based on cDNA sequences.

Bands amplified by standard and RT-PCR were cloned using the TopoTA vector (Invitrogen) under blue-white and kanamycin selection. White colonies were screened via a PCR-stab technique described [75] with vector-specific primers. Plasmids were isolated from clones containing correct-sized inserts using QIAprep Spin Miniprep kit (Qiagen), sequenced in complementary directions through ABI Big-Dye technology (Applied Biosystems, Foster City, CA) and analyzed with Sequencher 4.0 (Gene Codes Corporation, Ann Arbor, MI).

### Phylogenetic analyses

Inferred RPB1 amino acid sequences from *Glaucocystis *[DQ223185] and *Cyanophora *[DQ223186] were aligned with a data set of RPB1 sequences from organisms present in GenBank and genome-sequencing databases (see Additional file 1). Sequences through the conserved H region [30] were aligned with CLUSTAL X [76], and adjusted by eye. Areas of the sequences with gaps that could not be placed with confidence were excluded from the alignment. Two separate data sets were analyzed. One included 47 representatives from the broadest diversity of sequences available; this alignment including a partial sequence from *Cyanophora *(regions A-G). A second smaller alignment, representing 30 taxa, was constructed by removing sequences with large amounts of missing data, as well as sequences demonstrated to produce phylogenetic artifacts in previous analyses.

Maximum-likelihood parameters (amino acid frequencies, percent invariant sites, and α for modeling rate variation among sites) were estimated in TREEPUZZLE 5.0 [77] under a Jones-Taylor-Thornton (JTT [78]) substitution matrix with invariable + Γ (four category) distribution of rates. Maximum-likelihood trees were recovered in ProtML (Phylip 3.6 [79]), using the parameters determined in TREEPUZZLE and 10 random sequence addition searches with global rearrangements. One hundred likelihood bootstrap replicates were performed under a JTT + uniform rate model, with 5 random sequence additions per replicate and global rearrangements.

Analyses were performed using MRBAYES 3.1 [80], with the same parameters used with ML, to determine the consensus Bayesian tree and to assess strength of support for tree nodes. Two simultaneous runs were performed, each with four chains (one cold), for one million generations, and trees were sampled every 100 generations. The "burn-in" required to converge on stable likelihood values was determined empirically, and trees sampled during the burn-in were eliminated prior to computing the 50% majority-rule consensus tree.

One thousand distance bootstrap replicates also were run using in PROTDIST and NEIGHBOR (Phylip 3.6), with a JTT substitution matrix. Parsimony bootstrap was carried out in PAUP [81] with 1000 replicates and 20 random sequence edition per replicate. Certain *a priori *phylogenetic hypotheses were examined with *RPB*1 data by implementing the Kishino-Hasegawa (KH), as well as the more conservative Shimodaira-Hasegawa (SH) tests [82,83] in PROTML (Phylip 3.6).

### Analyses of long-branch indicators

To assess the bases for the overall topology of the *RPB*1 tree, and specific differences between that topology and trees recovered from other data sets, we analyzed "long-branch" tendencies of sequences in the 30 taxon data set. We used three different methods, each independent of *a priori *assumptions about relationships among distinct eukaryotic lineages. 1) A χ^2 ^test was performed in TREEPUZZLE to ascertain which sequences deviated significantly from average amino acid composition. 2) Unique autapomorphies at otherwise highly conserved sites were scored for all individual sequences, using MACCLADE 3.06 [84]. Unique substitutions were counted at sites that were invariable in all but one or two sequences, that is, sites clearly under strong stabilizing selection but still capable of at least some change. If two changes were present for a given character, they were scored only if unequivocally discrete substitutions; that is, each was a different residue or they occurred independently in taxa that could not be related evolutionarily. 3) One hundred randomized sequences were constructed in MCCLADE 3.06, composed of the average amino acid frequencies calculated in TREEPUZZLE. These sequences were added individually to the *RPB*1 alignment and used in parsimony analyses with 20 random sequence additions to determine the empirical tendency of each *RPB*1 sequences to attract "long-branches." Sequences were deemed to be prone to long-branch artifacts if they attracted a random sequence in 5% or more of parsimony replicates. These sequences were removed from the alignment, and the analyses repeated with three progressively smaller subsets of RPB1 genes with decreasing apparent long-branch tendencies. With the smallest of these sub-alignments (five taxa), 1000 bootstrap replicates were performed with each of 10 random sequences (20 random additions each), to determine the distribution of their points of attachment when sequences with stronger "long-branch" tendencies were removed.

## Authors' contributions

LH sequenced glaucocystophyte RPB1 genes and cDNA, performed bioinformatics searches for other eukaryotic sequences, annotated intron positions, and was primarily responsible for multiple sequence alignments. JWS performed analyses of long-branch indicators. Both authors contributed ideas contained in the paper, worked on phylogenetic analyses and contributed to authorship of the manuscript. Both authors read and approved the final manuscript.

## Supplementary Material

Additional File 1 Supplementary Table. Database sources for sequence used in this investigation Click here for file

Additional File 2 Bayesian inference tree. Consensus Bayesian tree inferred from the alignment of 30 RPB1 sequences. Branch lengths and posterior probabilities were recovered using the sumt command in MrBayes. See methods section and legend to figure 2 for additional details. Click here for file
