# Supplementary material for: The largest subunit of RNA polymerase II from the Glaucocystophyta: functional constraint and short-branch exclusion in deep eukaryotic phylogeny
Source: BMC Evol Biol. 2005 Dec 9;5:71. doi: 10.1186/1471-2148-5-71 (PMC1326215; doi:10.1186/1471-2148-5-71)
Supplement: Additional File 1 — Supplementary Table. Database sources for sequence used in this investigation [file 1471-2148-5-71-S1.pdf]

Supplementary Table. URL of database provided for unannotated sequences retrieved by Blast search.

Taxon	Accession or URL
Acanthamoeba castellanii	AAC18417
Arabidopsis thaliana	AL161588
Artemia salina	AAC83397
Bonnemaisonia hamifera	AAC18416
Botryocladia uvarioides	AAK00309
Caenorhabditis elegans	M29235
Candida albicans	EAL00529
Chlamydomonas reinhardtii	http://www.chlamy.org/
Coccidioides immitis	http://www.ncbi.nlm.nih.gov/sutils/genom_table.cgi
Coprinopsis cinerea	http://www.ncbi.nlm.nih.gov/sutils/genom_table.cgi
Crassostrea gigas	U10334
Cryptococcus neoformans	AE017345
Cryptosporidium parvum	XM_626822
Cyanidioschyzon merolae	AB095187
Cyanophora paradoxa	DG223186, DG223187
Dictyostelium discoideum	AAFI01000059
Drosophila melanogaster	AAA28868
Encephalitozoon cuniculi	AL590443
Entamoeba histolytica	AY623801
Giardia intestinalis	AB092412
Giardia lamblia	http://www.ncbi.nlm.nih.gov/sutils/genom_table.cgi
Gibberella zeae	XM_381092
Glaucocystis nostochinearum	DG183225
Glaucosphaera vacuolata	AAK00310
Helobdella stagnalis	AAA50227
Homo sapiens	X74874
Leishmania major	AF009163
Leptomonas seymouri	AF338253
Mastigamoeba invertens"	AF083338
Monoblepharis macrandra	AF315822
Monosiga brevicollis	AF315821
Naegleria gruberi	AAM45151
Neurospora crassa	XM_329292
Nosema locustae	AF061288
Oryza sativa	XM_493925
Plasmodium falciparum	X16561
Porphyra yezoensis	AAC17924.
Saccharomyces cerevisiae	X03128
Schizosaccharomyces pombe	X56564
Spirogyra sp.	U90210
Stylonychia mytilus	AF315823
Thalassiosira pseudonana	http://genome.jgi-psf.org/thaps1/thaps1.home.html
Theileria parva	XM_758110
Trichomonas vaginalis	U20501

Trypanosoma brucei	J04841
Ustilago maydis	XM_754917
Vairimorpha necatrix	AF060234
