# Supplementary material for: The largest subunit of RNA polymerase II from the Glaucocystophyta: functional constraint and short-branch exclusion in deep eukaryotic phylogeny
Source: BMC Evol Biol. 2005 Dec 9;5:71. doi: 10.1186/1471-2148-5-71 (PMC1326215; doi:10.1186/1471-2148-5-71)

Additional file 2. Bayesian tree with branch lengths and posterior probabilities shown above or to the right of their respective nodes.

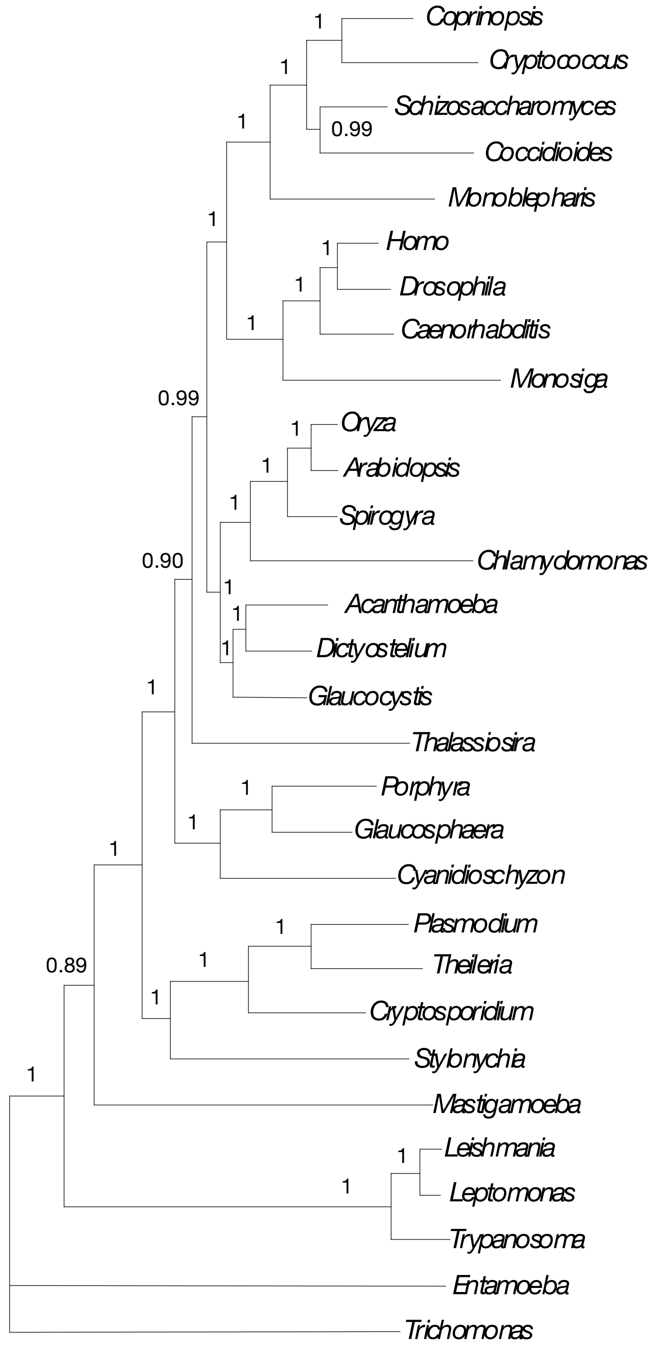

Supplement: Additional File 2 — Bayesian inference tree. Consensus Bayesian tree inferred from the alignment of 30 RPB1 sequences. Branch lengths and posterior probabilities were recovered using the sumt command in MrBayes. See methods section and legend to figure 2 for additional details. [file 1471-2148-5-71-S2.pdf]
